# Isolation of *Hafnia paralvei* co-harbouring *bla*_NDM-1_ and *bla*_VIM-1_ in a woman who underwent allogeneic hematopoietic stem cell transplantation

**DOI:** 10.1007/s15010-022-01976-8

**Published:** 2023-01-03

**Authors:** Tobias Manuel Appel, Claudia Stein, Christian Brandt, Jürgen Rödel, Jochen J. Frietsch, Jenny Miethke, Andreas Hochhaus, Inken Hilgendorf

**Affiliations:** 1 Klinik für Innere Medizin II, Abteilung für Hämatologie und internistische Onkologie, Universitätsklinikum Jena, Kastanienstraße 1, 07747 Jena, Germany; 2 Institut für Infektionsmedizin und Krankenhaushygiene, Universitätsklinikum Jena, Kastanienstraße 1, 07747 Jena, Germany; 3 Institut für Medizinische Mikrobiologie, Universitätsklinikum Jena, Kastanienstraße 1, 07747 Jena, Germany; 4 Medizinische Klinik 2, Infektiologie, Universitätsklinikum Frankfurt, Theodor-Stern-Kai 7, 60590 Frankfurt am Main, Germany

**Keywords:** Antimicrobial drug resistance, Beta-lactam resistance, Carbapenem-resistant enterobacteriaceae, Colistin, Fosfomycin

## Abstract

Metallo-*β*-lactamases (MBL) are a threat to public health, since they dramatically limit the use of *β*-lactams. We report the isolation of a multidrug-resistant *Hafnia paralvei* strain from urine and a rectal swab of a female patient after allogeneic hematopoietic stem cell transplantation for myelodysplastic syndrome. Antimicrobial susceptibility testing yielded resistance to trimethoprim/sulfamethoxazole, colistin, fosfomycin and all *β*-lactams, except cefiderocol. Whole genome sequencing revealed the presence of plasmid-encoded NDM-1 and VIM-1 carbapenemases. This finding highlights the importance of epidemiological surveillance and new therapeutic options for MBL.

## Introduction

Antimicrobial resistance is a threat to global public health with an estimated 4.95 million associated deaths in 2019 worldwide [1]. Carbapenemase-producing Enterobacterales (CPE) are of special concern, since they drastically reduce the efficacy of *β*-lactams, the cornerstone of modern antimicrobial therapy. Although new *β*-lactam/*β*-lactamase inhibitor combinations to target class A and several class D carbapenemases have been released in the last decade, specific inhibitors for class B carbapenemases are still lacking. Herein, we report the case of a patient carrying a multidrug resistant *Hafnia paralvei* strain co-harbouring *bla*_NDM-1_ and *bla*_VIM-1_.

## Case report

The 59-year-old woman underwent allogeneic hematopoietic stem cell transplantation (SCT) after reduced toxicity conditioning with treosulfan, fludarabine and antithymocyte globulin for treatment of myelodysplastic syndrome with high-risk features. In the six months prior to SCT, the patient had been treated with several antimicrobials including amoxicillin/clavulanic acid, ciprofloxacin, trimethoprim/sulfamethoxazole, linezolid, isavuconazole, posaconazole, fluconazole, acyclovir either as treatment of bloodstream infections (BSI), as well as a pulmonary fungal disease, or as prophylaxis during neutropenia.

On day two before SCT the patient developed fever and was found to be hypotensive, requiring vasopressor support with epinephrine. Due to previous BSI with *Citrobacter freundii* and *Enterococcus faecium* empirical anti-infective therapy with intravenous meropenem 2 g every 8 h and teicoplanin with a starting dose of 800 mg and a maintenance dose of 400 mg every 24 h was started. On day four, teicoplanin was switched to tigecycline 50 mg every 12 h and isavuconazole 200 mg once daily was added since the patient continued to require vasopressor support. Afterwards, epinephrine was reduced gradually and suspended after 6 days. In peripheral blood cultures growth of vancomycin-susceptible *Enterococcus faecium* was detected. On day 12 meropenem was de-escalated to ceftazidime 2 g every 8 h. The patient fully recovered from the infection after 17 days of antimicrobial therapy. However, on day 22 after SCT she complained about abdominal cramps, nausea and urinary retention. Ultrasound examination of the bladder revealed a residual volume of 135 ml; therefore, a urinary catheter was placed and a urine sample was taken. The following day the patient continued with abdominal complaints and fever (38.4 °C). Empirical treatment with meropenem 2 g every 8 h was reinitiated subsequently. Three days later, the patient was afebrile and the abdominal complaints resolved. Due to clinical resolution and normal levels of C reactive protein, treatment with meropenem was discontinued.

Further urinary and blood cultures remained negative, and the patient was discharged three weeks later after resolution of neutropenia. Before the discharge to a rehabilitation facility, a rectal swab for the screening of colonization with multidrug-resistant bacteria was taken, which yielded intestinal colonization with *H. alvei* with the same susceptibility profile as the reported below.

For microbiological diagnostics the urine sample was streaked onto Columbia sheep blood agar (BD, Heidelberg, Germany) and Drigalski lactose agar (Oxoid, ThermoFisher Scientific, Wesel, Germany) for overnight incubation at 37 °C. Colonies were identified as *Hafnia alvei* by Vitek MS (bioMérieux, Nürtingen, Germany). Antimicrobial susceptibility testing (AST) was performed by determination of minimum inhibitory concentrations (MIC) using Vitek 2 (bioMérieux) and the MICRONAUT-S MDR MRGN Screening microtiter system (Merlin Diagnostika, Bornheim, Germany; distributed by Sifin Diagnostics, Berlin, Germany). The MIC for cefiderocol was determined by a test strip (Liofilchem, Roseto degli Abruzzi, Italy; distributed by Bestbion, Köln, Germany). Breakpoints were interpreted according to European Committee on Antimicrobial Susceptibility Testing (EUCAST) criteria. The isolate was tested for *β*-lactamase genes by the eazyplex^®^ SuperBug CRE and AmpC assays (Amplex Diagnostics, Gars-Bahnhof, Germany).

With the exception of cefiderocol, AST yielded resistance to all tested *β*-lactams, fosfomycin, colistin and trimethoprim/sulfamethoxazole (Table 1). Furthermore, the isolate was tested positive for VIM and NDM carbapenemases and harboured the AmpC *β*-lactamase ACC-1, which is intrinsic in species of *Hafnia*.

**Table 1:** Antimicrobial susceptibility testing of the isolated *H. paralvei* strain collected from urine

Antibiotic	MIC (mg/L)	Category^a^
Piperacillin/tazobactam	> 64/4	R
Cefotaxime	> 2	R
Ceftazidime	> 128	R
Ceftazidime/avibactam	> 16/4	R
Ceftolozane/tazobactam	> 8/4	R
Meropenem	> 128	R
Imipenem	> 8	R
Cefiderocol	1	S
Fosfomycin	> 128	R
Levofloxacin	1	I
Amikacin	4	S
Trimethoprim/sulfamethoxazole	> 4/76	R
Tigecycline	0.5	N/A^b^
Colistin	8	R
Chloramphenicol	≤ 8	S

*MIC* Minimum inhibitory concentration

^a^The breakpoints to discriminate between susceptible (S), susceptible with increased exposure (I), and resistant (R) were interpreted according to the EUCAST guideline

^b^*N/A* not available

The isolate was sequenced on an Oxford Nanopore Technology device. DNA was extracted with the ZymoBIOMICS™ DNA Microprep kit (ZYMO Research) as well as the SQK-LSK-110 library kit (Oxford Nanopore Technologies) according to the manufacture’s recommendations. For sequencing, the library was loaded on a FLO-MIN105 version R9.4 flow cell. Taxonomic classification was done with GTDB-Tk version 1.6.0 software toolkit and the GTDB database version r202. The sequence data has been deposited at the NCBI BioProject database (BioProject ID PRJNA826682).

The bacterial genome showed the highest similarity (99.41%) with the *Hafnia paralvei* ATCC 29,927 reference genome (NCBI database GCF_001655005.1). Furthermore, four plasmids with sufficient coverage were detected: a 47 kb plasmid (incompatibility (Inc) group IncU_1) carrying *bla*_VIM-1_, a 41 kb plasmid (IncN2_1) harbouring *bla*_NDM-1_ in addition to a 10 kb and a 40 kb plasmid without carbapenemase-encoding genes. The VIM-1 encoding gene was located on a class 1 integron together with additional antimicrobial resistance genes, including *sul1*, which is able to confer resistance to aminoglycosides, phenicol and sulphonamides. The 41 kb plasmid harbouring *bla*_NDM-1_ was highly similar (99.94%) to the plasmid pCRE1.4, which was isolated from *Escherichia coli* and is available in the NCBI database (accession number CP034403). *bla*_NDM-1_ was flanked by two IS elements (ISEc33, ISSen4 family transposase), as well as the bleomycin resistance protein encoding gene *ble*_MBL_ downstream. As expected, the AmpC β-lactamase encoding gene *bla*_ACC-1_ was located on the chromosome, while quinolone antibiotic resistance genes (*qnrS*) were found both on the chromosome and on plasmids. Common fosfomycin and colistin resistance determinants, such as *fos*A/B/C and *mcr*, respectively, were not detected.

## Discussion

*H. paralvei* usually carries a chromosomally encoded, inducible class C *β*-lactamase ACC-1, which can confer resistance to a broad range of *β*-lactams [2]. Additional changes in the outer membrane, such as loss of function of porins, can lead to carbapenem resistance, even in the absence of carbapenemases [3]. Thus, the remaining therapeutical *β*-lactam armamentarium is limited. Furthermore, metallo-*β*-lactamases (MBLs) are able to hydrolyse all bicyclic *β*-lactams, and therefore, are a mayor threat to global public health [4]. The production of both a serine- and a MBL virtually excludes *β*-lactams as a single therapeutic agent.

Co-production of multiple carbapenemases has been reported increasingly in *Pseudomonas* spp. and *Acinetobacter* spp. and less frequently in Enterobacterales, in particular in *K. pneumoniae* [5]. Both NDM-1 and VIM-1 are mainly plasmid-mediated group 1 MBLs, which have been described mostly in Enterobacterales [4]. To our knowledge, neither NDM-, nor VIM-encoding genes have been reported previously in species of *Hafnia*.

The association between *bla*_NDM-1_ and *ble*_MBL_ has been described previously in Enterobacterales and *A. baumannii*, in which both genes were found to be configurated in an operon [6]. In vitro, BRP_MBL_, the *ble*_MBL_ encoded protein, reduced mutation rates in hypermutable *E. coli*, and was postulated to stabilize *bla*_NDM-1_. Dortet et al. further characterized the *bla*_NDM-1_-*ble*_MBL_-operon and reported the binding and sequestration of bleomycin-like molecules by BRP_MBL_ and thereby preventing DNA damage [7].

Jayol et al. found high rates of colistin resistance in isolates of *H. alvei* and *H. paralvei* and described a close phylogenetic relationship to naturally colistin-resistant genera, such as *Proteus* spp., *Providencia* spp. and *Morganella* spp. Thus, the authors concluded that *Hafnia* spp. should be considered naturally colistin-resistant as well [8]. Similarly, Stock et al. reported resistance to fosfomycin in 97.3% of clinical *H. alvei* isolates collected in the early 1990s and therefore, suggested fosfomycin resistance to be natural in this specie [9]. However, the underlying mechanisms of resistance have not been established yet. Both findings might explain resistance to fosfomycin and colistin, in the absence of common resistance-determinants.

Regarding the patient, the resolution of symptoms under therapy with meropenem and the absence of significant levels of markers of systemic inflammation calls into question the clinical significance of the reported microbiological finding. However, the isolation of *H. paralvei* co-harbouring *bla*_NDM-1_ and *bla*_VIM-1_ points out the possible role of *Hafnia* spp. as a potential reservoir of carbapenem resistance along with other Enterobacterales.

Species of *Hafnia* are part of the microbiota of many mammals, including humans. There are several case reports of patients with extraintestinal *H. alvei-* and *H. paralvei*-infections available in the literature [10]. Most patients were of advanced age and/or severely immunosuppressed. Interestingly, in several case reports, the isolation of species of *Enterococcus* and *H. alvei*/*H. paralvei* was reported. Further studies are required to elucidate the question, whether there is an association between species of both genera.

This report underlines the importance of epidemiological surveillance, efforts to prevent the spread of antimicrobial resistance and the development of further therapeutic options for the treatment of patients with infections caused by MBL-producing microorganisms.
